# Supplementary material for: Metformin Induces Resistance of Cancer Cells to the Proteasome Inhibitor Bortezomib
Source: Biomolecules. 2022 May 28;12(6):756. doi: 10.3390/biom12060756 (PMC9221333; doi:10.3390/biom12060756)
Supplement: Supplementary file 1 [file biomolecules-12-00756-s001.zip › biomolecules-1717160-supplementary - update 6.1/Supplementary Figure.pdf]

Figure S1. Original Western blots and densitometric analysis thereof.

H1299 Western Blot A

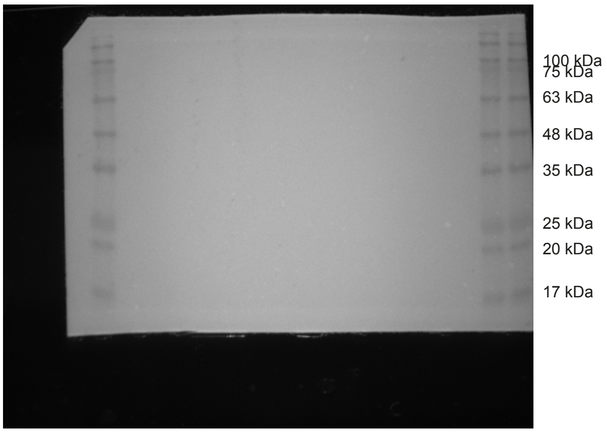

stainfree membrane

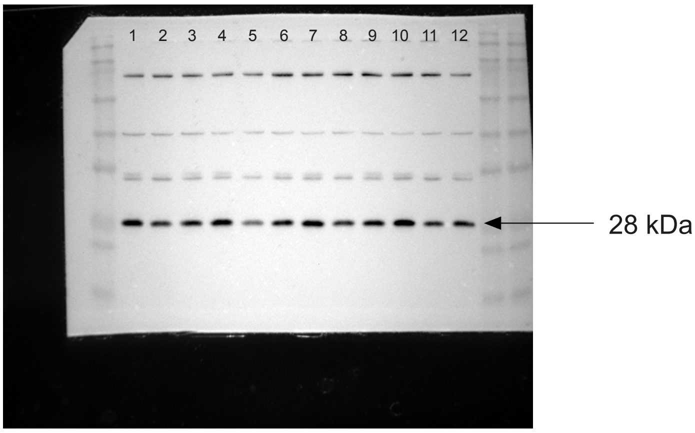

p28 staining

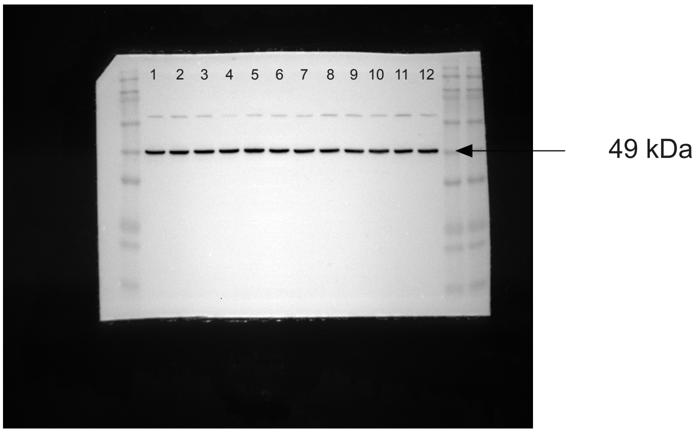

Rpn6 staining

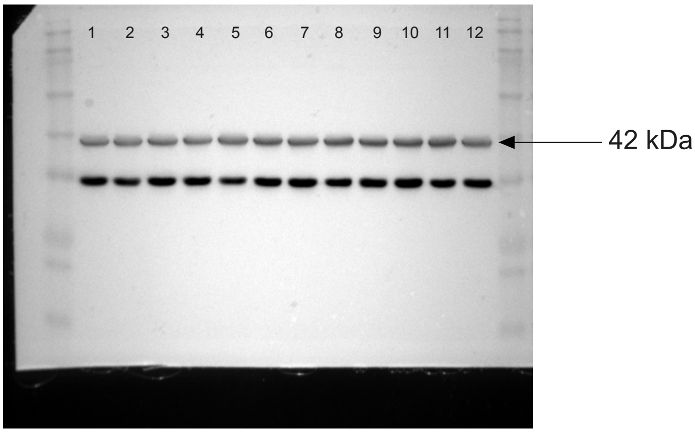

Beta-actin staining

H1299 Western Blot A

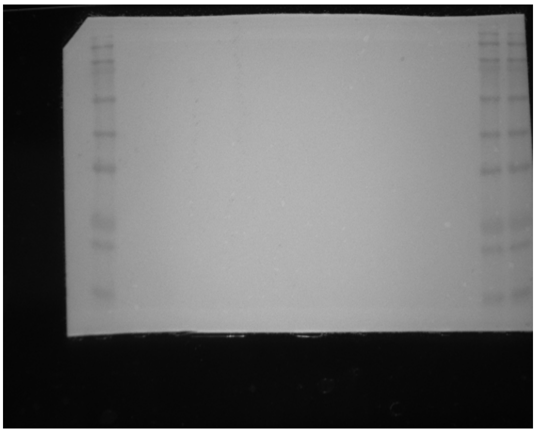

stainfree membrane

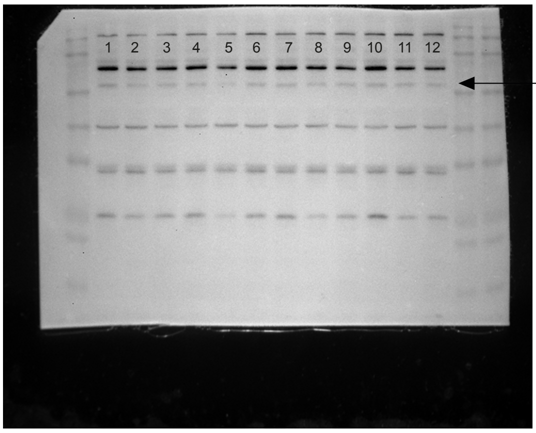

p70-phospho-S6kinase

H1299 Western Blot B

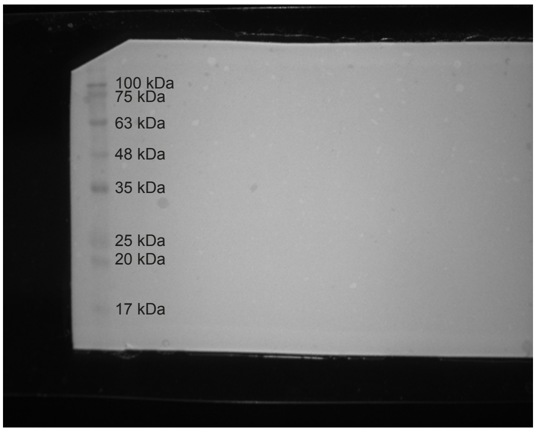

stainfree membrane

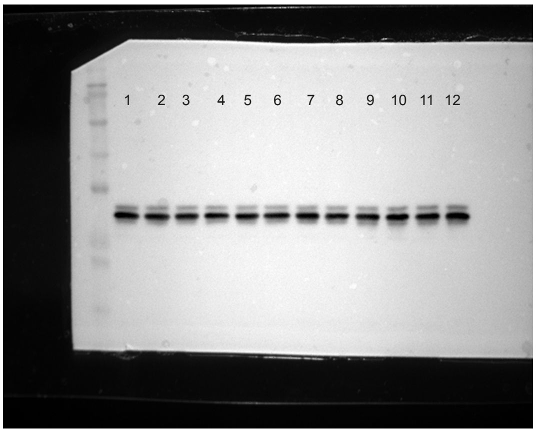

3 bands around 30 kDA

Alpha 1-7 staining

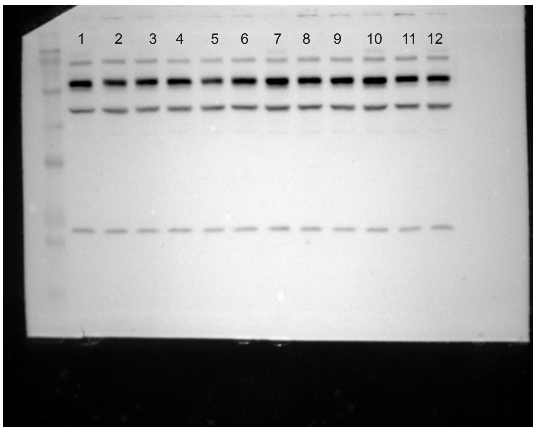

56 kDa

S5b staining

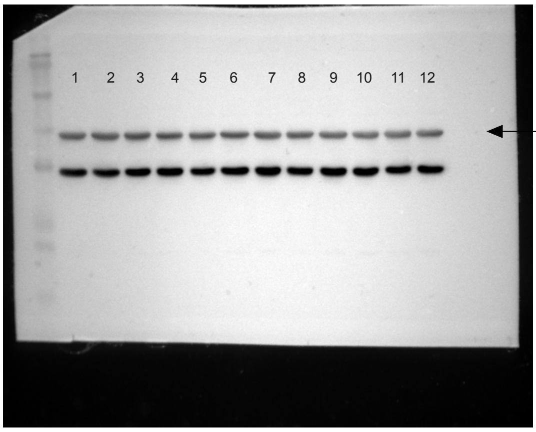

42 kDa

Beta-actin staining

H1299 Western Blot B

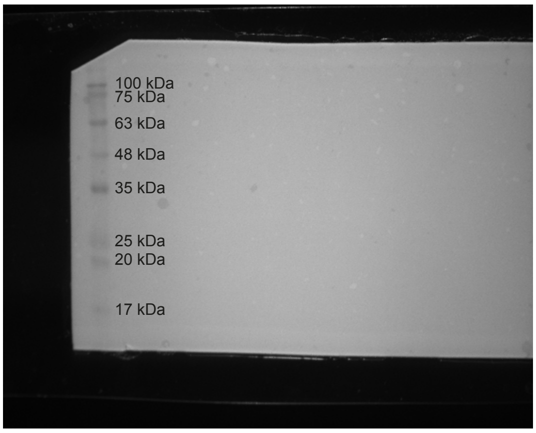

stainfree membrane

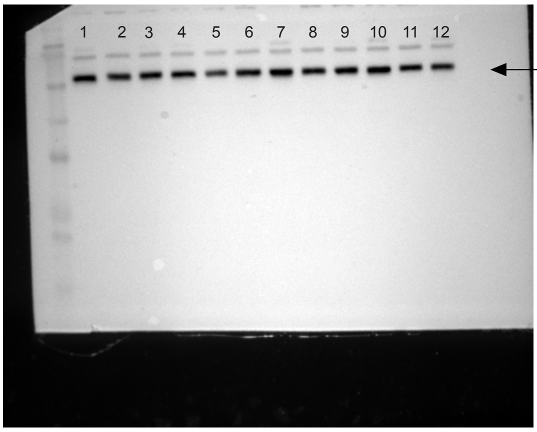

70 kDa

total p70 S6kinase

Jeko-1-Western Blot A

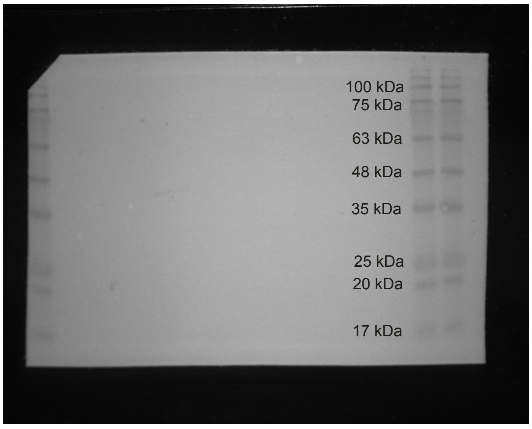

stainfree membrane

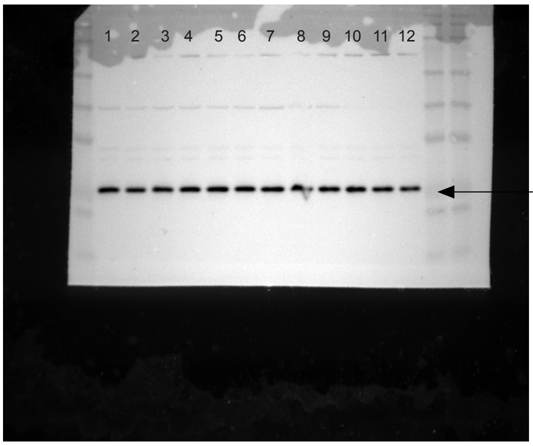

28 kDa

p28 staining

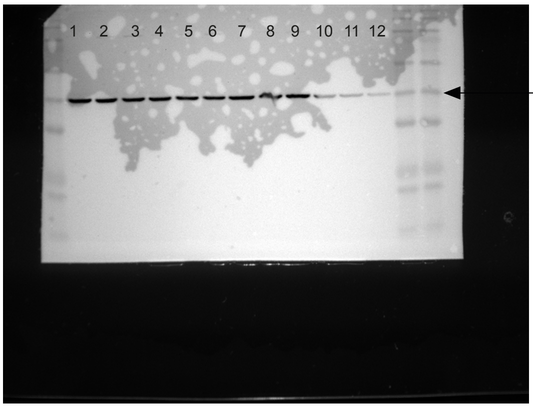

49 kDa

Rpn6 staining

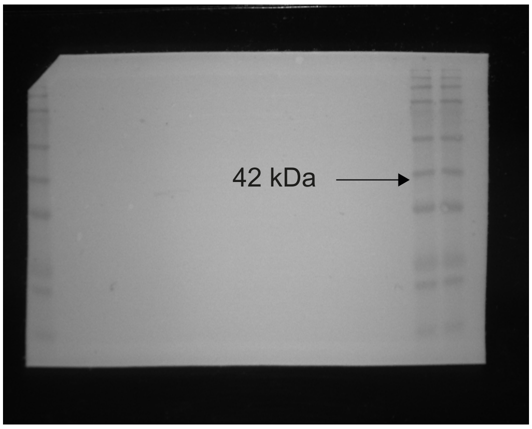

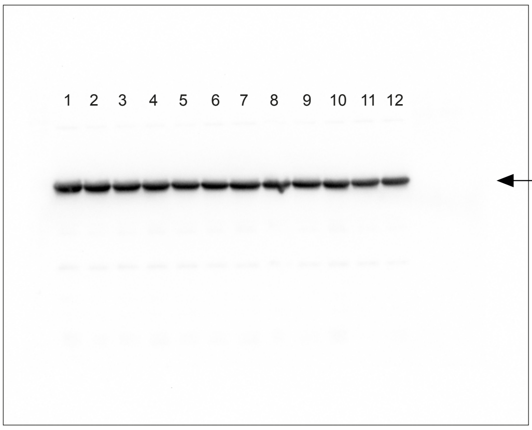

Beta-actin staining

Jeko-1-Western Blot A

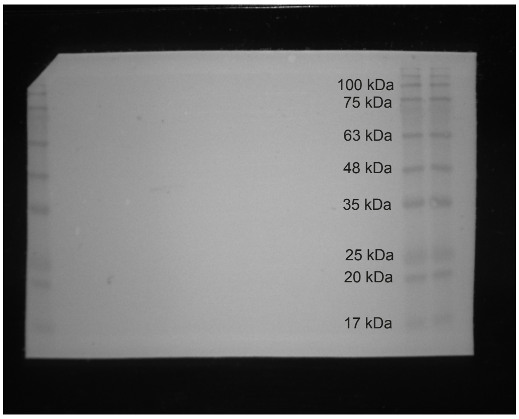

stainfree membrane

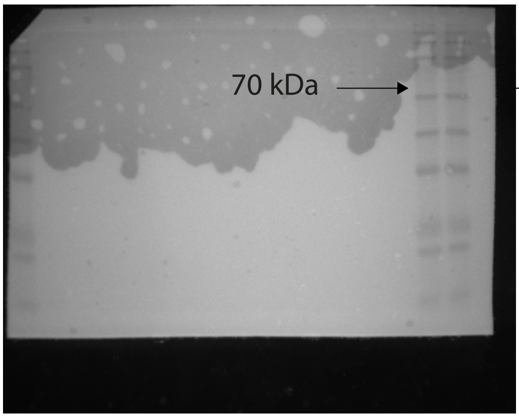

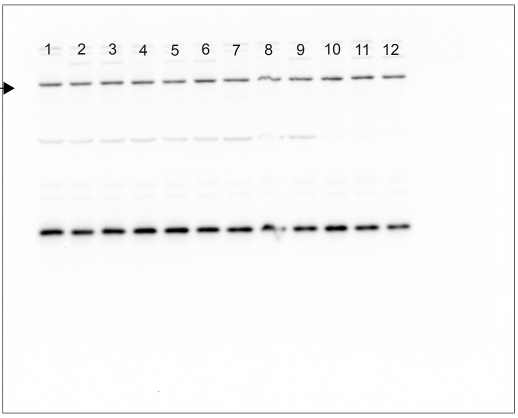

phospho-p70-S6kinase

Jeko-1-Western Blot B

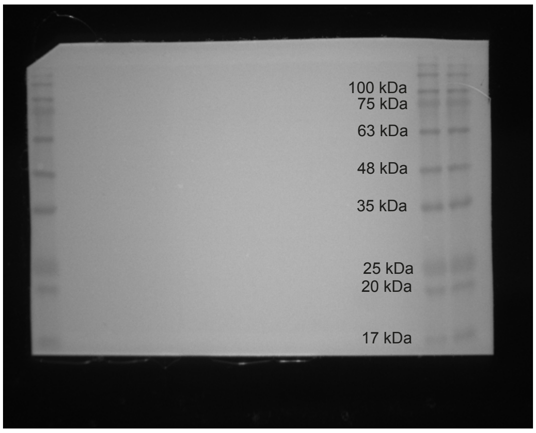

stainfree membrane

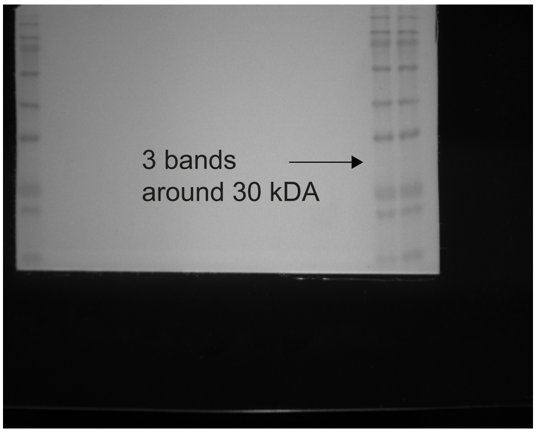

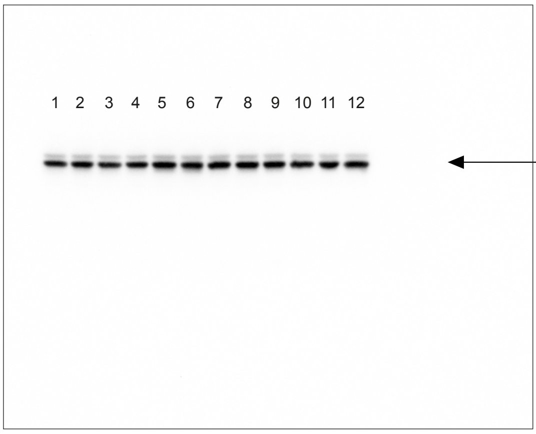

Alpha 1-7 staining

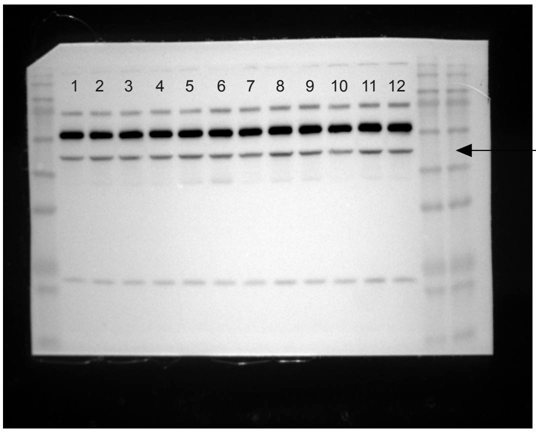

56 kDa

S5b staining

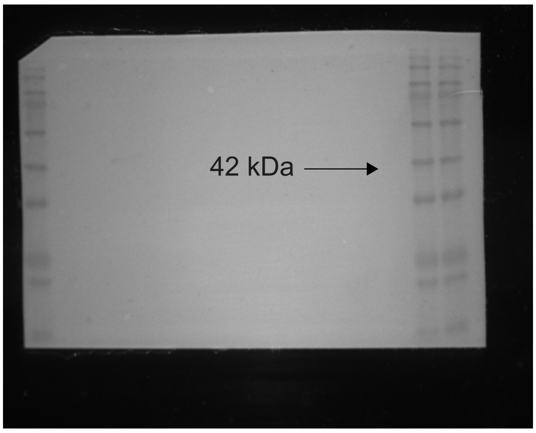

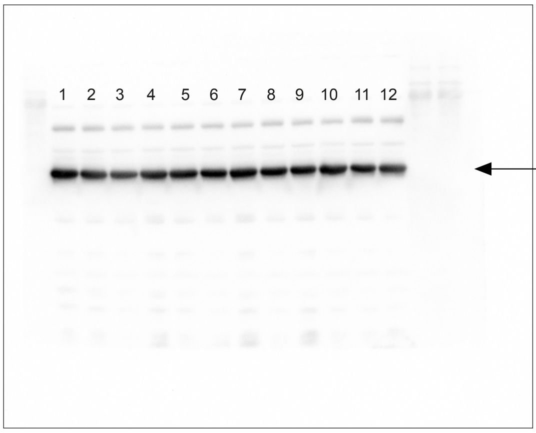

Beta-actin staining

Jeko-1-Western Blot B

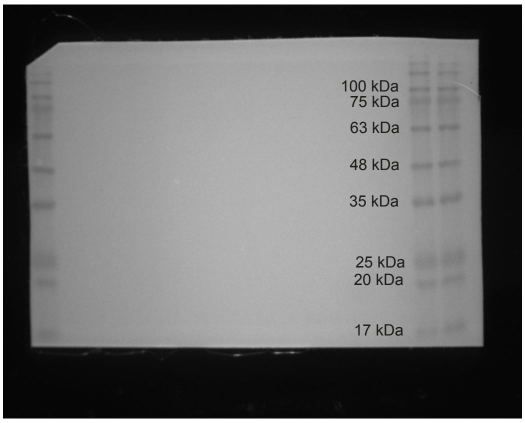

stainfree membrane

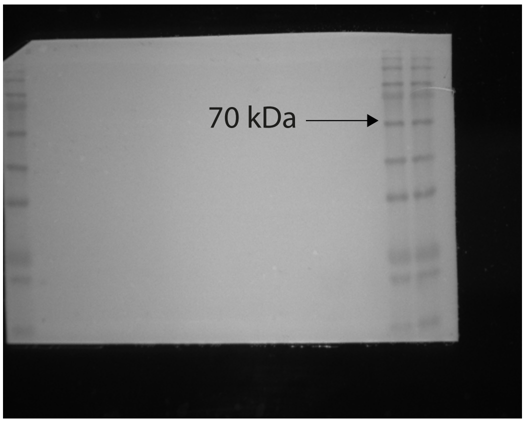

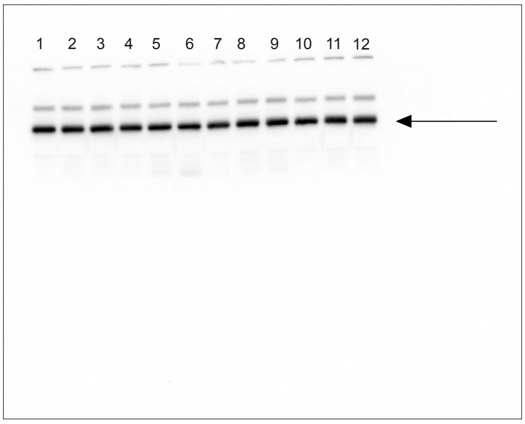

total p70 S6kinase

Western Blot A-H1299

p28

Exp. No.	Lane	Treatment	Adj. Volume
1	1	control	4178583
	2	metformin	2642380
	3	metformin+pyruvate	3064778
2	4	control	4028724
	5	metformin	1746210
	6	metformin+pyruvate	3131670
3	7	control	4211032
	8	metformin	2638216
	9	metformin+pyruvate	3413213
4	10	control	4853005
	11	metformin	2402062
	12	metformin+pyruvate	3008394

Rpn6

Exp. No.	Lane	Treatment	Adj. Volume
1	1	control	4709918
	2	metformin	5107653
	3	metformin+pyruvate	5065431
2	4	control	5068593
	5	metformin	5775579
	6	metformin+pyruvate	4896690
3	7	control	4919968
	8	metformin	4525260
	9	metformin+pyruvate	4148460
4	10	control	4463648
	11	metformin	4454730
	12	metformin+pyruvate	4827584

p70-phospho-S6kinase

Exp. No.	Lane	Treatment	Adj. Volume
1	1	control	402636
	2	metformin	280868
	3	metformin+pyruvate	340984
2	4	control	317640
	5	metformin	254212
	6	metformin+pyruvate	350001
3	7	control	343969
	8	metformin	312960
	9	metformin+pyruvate	336930
4	10	control	435180
	11	metformin	304890
	12	metformin+pyruvate	337386

Western Blot B-H1299

S5b

Exp. No.	Lane	Treatment	Adj. Volume
1	1	control	4994616
	2	metformin	5356893
	3	metformin+pyruvate	4854020
2	4	control	5278667
	5	metformin	5304992
	6	metformin+pyruvate	5824170
3	7	control	6307322
	8	metformin	5645038
	9	metformin+pyruvate	5565270
4	10	control	5802630
	11	metformin	5643840
	12	metformin+pyruvate	5410151

alpha 1-7

Exp. No.	Lane	Treatment	Adj. Volume
1	1	control	12939210
	2	metformin	11698592
	3	metformin+pyruvate	10328010
2	4	control	10449384
	5	metformin	10287908
	6	metformin+pyruvate	10968451
3	7	control	11154270
	8	metformin	10259910
	9	metformin+pyruvate	11438368
4	10	control	12293361
	11	metformin	12863808
	12	metformin+pyruvate	14033440

total p70S6kinase

Exp. No.	Lane	Treatment	Adj. Volume
1	1	control	7304259
	2	metformin	6275513
	3	metformin+pyruvate	6650420
2	4	control	6828990
	5	metformin	4626692
	6	metformin+pyruvate	7219094
3	7	control	7873800
	8	metformin	6336555
	9	metformin+pyruvate	6854910
4	10	control	7761450
	11	metformin	5938591
	12	metformin+pyruvate	5592307

Western Blot A-H1299
beta actin (loading control)

Exp. No.	Lane	Treatment	Adj. Volume
1	1	control	7026600
	2	metformin	7152382
	3	metformin+pyruvate	7940650
2	4	control	7283202
	5	metformin	8800373
	6	metformin+pyruvate	9172218
3	7	control	9046327
	8	metformin	9623330
	9	metformin+pyruvate	9059843
4	10	control	9503938
	11	metformin	9536430
	12	metformin+pyruvate	8078720

Western Blot B-H1299
beta actin (loading control)

Exp. No.	Lane	Treatment	Adj. Volume
1	1	control	7280100
	2	metformin	8173336
	3	metformin+pyruvate	8366500
2	4	control	8297863
	5	metformin	8000640
	6	metformin+pyruvate	8766816
3	7	control	8375301
	8	metformin	7828771
	9	metformin+pyruvate	7261440
4	10	control	6476910
	11	metformin	6886743
	12	metformin+pyruvate	6962786

Western Blot A-Jeko-1

p28

Exp. No.	Lane	Treatment	Adj. Volume
1	1	control	7012608
	2	metformin	5799900
	3	metformin+pyruvate	6420960
2	4	control	6942078
	5	metformin	7367150
	6	metformin+pyruvate	6751590
3	7	control	7319136
	8	metformin	7070810
	9	metformin+pyruvate	6467490
4	10	control	6669043
	11	metformin	5914927
	12	metformin+pyruvate	5151735

Rpn6

Exp. No.	Lane	Treatment	Adj. Volume
1	1	control	4570038
	2	metformin	4588380
	3	metformin+pyruvate	3972805
2	4	control	3990134
	5	metformin	3795744
	6	metformin+pyruvate	3321247
3	7	control	4606805
	8	metformin	4141824
	9	metformin+pyruvate	4546773
	10		
	11		
	12		

p70-phospho-S6kinase

Exp. No.	Lane	Treatment	Adj. Volume
1	1	control	1708470
	2	metformin	1507044
	3	metformin+pyruvate	1774800
2	4	control	1751932
	5	metformin	1580370
	6	metformin+pyruvate	1836594
3	7	control	1806587
	8	metformin	1400332
	9	metformin+pyruvate	1651530
4	10	control	1850160
	11	metformin	1807410
	12	metformin+pyruvate	1556310

Western Blot B-Jeko-1

S5b

Exp. No.	Lane	Treatment	Adj. Volume
1	1	control	2947950
	2	metformin	3709057
	3	metformin+pyruvate	3229005
2	4	control	3590896
	5	metformin	4356988
	6	metformin+pyruvate	4069494
3	7	control	3677550
	8	metformin	4941927
	9	metformin+pyruvate	4196250
4	10	control	3450114
	11	metformin	4607694
	12	metformin+pyruvate	4355472

alpha 1-7

Exp. No.	Lane	Treatment	Adj. Volume
1	1	control	9094346
	2	metformin	10353024
	3	metformin+pyruvate	9164468
2	4	control	9414870
	5	metformin	12593967
	6	metformin+pyruvate	11375430
3	7	control	11513679
	8	metformin	11964450
	9	metformin+pyruvate	10888378
4	10	control	9894656
	11	metformin	9115077
	12	metformin+pyruvate	10840401

total p70S6kinase

Exp. No.	Lane	Treatment	Adj. Volume
1	1	control	7383090
	2	metformin	7281218
	3	metformin+pyruvate	7656580
2	4	control	6822930
	5	metformin	6892890
	6	metformin+pyruvate	6741074
3	7	control	6468801
	8	metformin	7294982
	9	metformin+pyruvate	7151142
4	10	control	7006139
	11	metformin	7187969
	12	metformin+pyruvate	7122467

**Western Blot A-Jeko-1**<br>***beta actin (loading control)***			
Exp. No.	Lane	Treatment	Adj. Volume
1	1	control	13724832
	2	metformin	12891210
	3	metformin+pyruvate	12719040
2	4	control	12470624
	5	metformin	11480416
	6	metformin+pyruvate	10998304
3	7	control	12194820
	8	metformin	11631978
	9	metformin+pyruvate	11038863
4	10	control	10451619
	11	metformin	9416839
	12	metformin+pyruvate	9165460

**Western Blot B-Jeko-1**<br>***beta actin (loading control)***			
Exp. No.	Lane	Treatment	Adj. Volume
1	1	control	13711040
	2	metformin	11092770
	3	metformin+pyruvate	9146147
2	4	control	11196090
	5	metformin	12738112
	6	metformin+pyruvate	12560910
3	7	control	13878235
	8	metformin	12896288
	9	metformin+pyruvate	11836823
4	10	control	13031488
	11	metformin	10760880
	12	metformin+pyruvate	10071435
