# Supplementary material for: Metformin Induces Resistance of Cancer Cells to the Proteasome Inhibitor Bortezomib
Source: Biomolecules. 2022 May 28;12(6):756. doi: 10.3390/biom12060756 (PMC9221333; doi:10.3390/biom12060756)
Supplement: Supplementary file 1 [file biomolecules-12-00756-s001.zip › biomolecules-1717160-supplementary - update 6.1/Supplementary Table S1.pdf]

Supplementary Table S1:

Overview on the clinical trials involving metformin and proteasome inhibitors (taken from clinicaltrials.gov, May 13th, 2022)

**Title**	**identifier**	**link**
Ongoing and completed studies on metformin and cancer (Mostly Breast, Prostate, Endometrial, Bladder, Colorectal, Lung cancer)		
**Investigation of Metformin for the Prevention of Progression of Precursor Multiple Myeloma** (Start: April 27, 2021, estimated end: January 31, 2023) Recruiting	NCT04850846	https://clinicaltrials.gov/ct2/show/NCT04850846.
**Metformin and Carbohydrate Restriction With Platinum Based Chemotherapy in Stage IIIB/IV Non-Squamous Non-Small Cell Lung Cancer (NS-NSCLC) (METRO)** (Start: December 2013; End: December 2016). Terminated	NCT02019979	https://clinicaltrials.gov/ct2/show/NCT02019979?term=metformin&cond=Cancer&draw=8&rank=68.
**Phase II Lung Metocore – Preoperative Metformin for Lung Cancer (Metocore)** (Start: December 5, 2016; End : July 2020) Completed	NCT03086733	https://clinicaltrials.gov/ct2/show/NCT03086733?term=metformin&cond=Cancer&draw=9&rank=77.
**Repurposing Metformin as Anticancer Drug: in Advanced Prostate Cancer** (Start: January 2017 ; End: January 2018).	NCT03137186	https://clinicaltrials.gov/ct2/show/NCT03137186?term=metformin&cond=Cancer&draw=2&rank=3.
**The Effect of Metformin on Breast Cancer Patients** (Start: June 1, 2019; Predicted End: September 15, 2020). Recruiting	NCT04559308;	https://clinicaltrials.gov/ct2/show/NCT04559308?term=NCT04559308&draw=2&rank=1

**Advanced Lung Cancer Treatment With Metformin and Chemo-Radiotherapy (ALMERA**) (Start: December 17, 2014; End: March 9, 2020) Terminated	NCT02115464	https://clinicaltrials.gov/ct2/show/NCT02115464?term=metformin&cond=Cancer&draw=3&rank=15.
Ongoing studies combining metformin and bortezomib (and nelfinavir)		
**Metformin, Nelfinavir, and Bortezomib in Treating Patients with Relapsed and/or Refractory Multiple Myeloma** (Start April 17, 2019 ; Estimated end August 21, 2022) Recruiting	NCT03829020	https://clinicaltrials.gov/ct2/show/NCT03829020).
